# Investigation of the Neuroprotective Impact of Nimodipine on Neuro2a Cells by Means of a Surgery-Like Stress Model

**DOI:** 10.3390/ijms151018453

**Published:** 2014-10-14

**Authors:** Eva Herzfeld, Christian Strauss, Sebastian Simmermacher, Kaya Bork, Rüdiger Horstkorte, Faramarz Dehghani, Christian Scheller

**Affiliations:** 1 Department of Neurosurgery, Martin-Luther University of Halle-Wittenberg, Ernst-Grube-Str. 40, 06120 Halle (Saale), Germany; E-Mails: christian.strauss@uk-halle.de (C.St.); sebastian.simmermacher@uk-halle.de (S.S.); christian.scheller@medizin.uni-halle.de (C.Sc.); 2 Institute for Physiological Chemistry, Martin-Luther University of Halle-Wittenberg, Hollystr. 1, 06114 Halle (Saale), Germany; E-Mails: kaya.bork@medizin.uni-halle.de (K.B.); ruediger.horstkorte@medizin.uni-halle.de (R.H.); 3 Department of Anatomy and Cell Biology, Martin-Luther University of Halle-Wittenberg, Große Steinstraße 52, 06108 Halle (Saale), Germany; E-Mail: faramarz.dehghani@medizin.uni-halle.de

**Keywords:** nimodipine, neuroprotection, Neuro2a, stress, surgery

## Abstract

Nimodipine is well characterized for the management of SAH (subarachnoid hemorrhage) and has been shown to promote a better outcome and less DIND (delayed ischemic neurological deficits). In rat experiments, enhanced axonal sprouting and higher survival of motoneurons was demonstrated after cutting or crushing the facial nerve by nimodipine. These results were confirmed in clinical trials following vestibular Schwannoma surgery. The mechanism of the protective competence of nimodipine is unknown. Therefore, in this study, we established an *in vitro* model to examine the survival of Neuro2a cells after different stress stimuli occurring during surgery with or without nimodipine. Nimodipine significantly decreased ethanol-induced cell death of cells up to approximately 9% in all tested concentrations. Heat-induced cell death was diminished by approximately 2.5% by nimodipine. Cell death induced by mechanical treatment was reduced up to 15% by nimodipine. Our findings indicate that nimodipine rescues Neuro2a cells faintly, but significantly, from ethanol-, heat- and mechanically-induced cell death to different extents in a dosage-dependent manner. This model seems suitable for further investigation of the molecular mechanisms involved in the neuroprotective signal pathways influenced by nimodipine.

## 1. Introduction

Nimodipine is a 1,4-dihydropyridine L-type calcium channel antagonist. It leads to an increase of cerebral blood flow by dilation of arterioles [1]. It is recommended for the management of aneurysmal subarachnoid hemorrhage (aSAH) and has a proven effect in reducing poor outcome and delayed ischemic neurological deficits (DIND) following aSAH [2,3]. Additionally, its beneficial effect in skull base, laryngeal and maxillofacial surgery has been demonstrated in several animal experiments [4,5] and clinical series [6,7,8]. These positive results have been attributed to neuroprotection [9]. In contrast, a randomized, double-blind, placebo-controlled trial of very early nimodipine treatment in stroke showed no beneficial effect [10]. However, the underlying cellular mechanisms remain in part unclear [9]. These observations raise the question of whether nimodipine is a reasonable addition to the routinely performed perioperative dexamethasone prophylaxis in neurosurgical interventions with high risk for postoperative neurological deterioration. In the presented study, surgery-associated stress, like heat or mechanical stress, has been mimicked. Bipolar coagulation and the use of a microscope [11] are intraoperative sources of possibly thermal impairment of nerve tissue. Tumor dissection may mechanically affect the surrounding intact nerve tissue. Coagulation of vessels may change the blood supply and the micro-environment. Besides mechanical stress, nerve tissue may be affected intraoperatively by an increase in temperature caused by the microscope and the bipolar coagulation or by dryness and ionic shifts, as well as by oxidative stress, which is represented by ethanol in this study. The present study was designed to analyze the direct neuroprotective effects of nimodipine on neuroblastoma Neuro2a cells after application of the abovementioned cell death inductors. We analyzed the neuroprotective effect of nimodipine on undifferentiated neuroblastoma Neuro2a cells, which were used to study neuronal differentiation, axonal growth and the signaling pathway [12]. Therefore, this neuroblastoma cell line represents PNS, as well as neurons [13]. 

## 2. Results

### 2.1. Lactate Dehydrogenase (LDH) Assay 

To analyze the cytotoxic effect of ethanol, nimodipine-treated and untreated cells were stressed as mentioned above, and the LDH release into the culture medium was measured. Total cell lysis served as the positive control.

**Figure 1:**
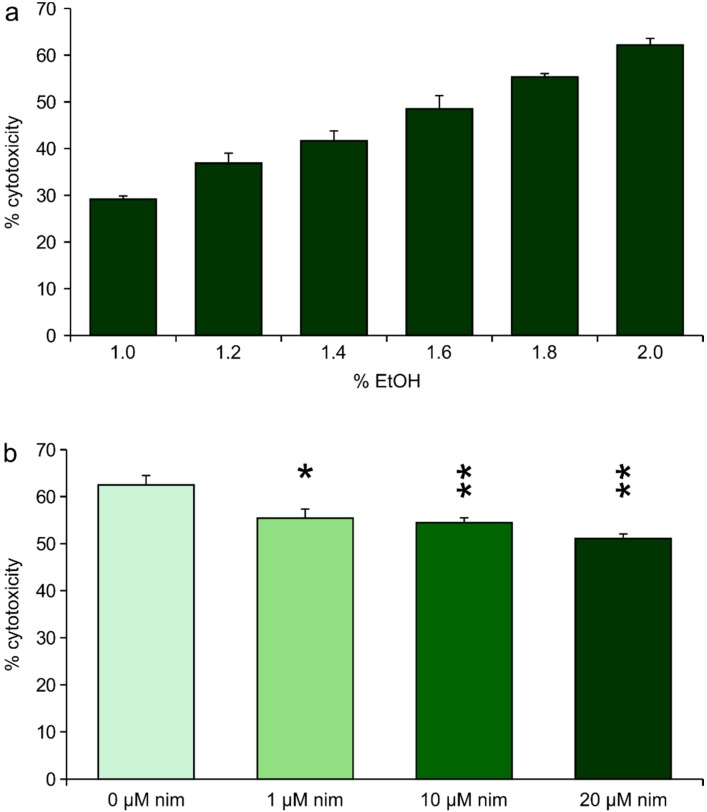
(**a**) Ratio of cytotoxicity induced by increasing ethanol concentrations. Values are given as the mean ± SD (error bars) of one representative out of at least three biologically independent experiments; and (**b**) LDH measurement after ethanol stress. Values are given as the mean ± SD. Nim: nimodipine; single asterisk: *p* ≤ 0.05 compared to non-treated cells; double asterisk: *p* ≤ 0.005 compared to non-treated cells.

In pre-experiments, we found 1.6% to 2.0% EtOH suitable to induce approximately 50% cytotoxicity, as shown in Figure 1a. Fifty percent cell death allows tracking of changes in both directions: higher and lower cell death. Therefore, we decided to use 1.8% EtOH to perform the experiments.

All tested nimodipine concentrations led to a reduced cytotoxicity when cells were challenged with 1.8% EtOH (*p* ≤ 0.05, Figure 1b). In detail, the treatment with 1, 10 or 20 μM nimodipine reduced the cytotoxicity of EtOH from 62% (untreated cells) to 55%, 54% and 51%. Similar results were measured for 1.6% EtOH (reduction from 61% to 55%, 54% and 55%, respectively; data not shown).

Osmotic stress was induced by treating the nimodipine pre-treated cells and untreated cells with NaCl concentrations between 100 and 200 mM. No significant changes in cytotoxicity were observed by increasing nimodipine concentrations (Figure S1).

**Figure 2:**
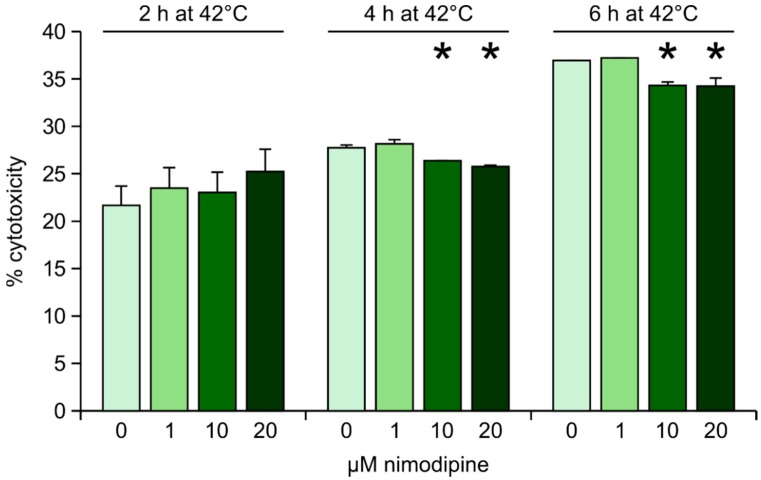
Lactate dehydrogenase (LDH) measurement after heat stress. Values are given as the mean ± SD (error bars) of one representative out of at least three biologically independent experiments. Nim: nimodipine; single asterisk: *p* ≤ 0.05 compared to non-treated cells.

Heat stress was induced by transferring the nimodipine pre-treated cells and the control cells to 42 °C for 2, 4 or 6 h, respectively. After heat incubation, cells were returned to 37 °C. No difference between nimodipine-treated and untreated cells was observed when cells were incubated at 42 °C for 2 h. When the cells were exposed to heat for 4 or 6 h, nimodipine concentrations of 10 and 20 μM, but not 1 μM, reduced cytotoxicity slightly, but significantly (*p* ≤ 0.05, Figure 2).

**Figure 3:**
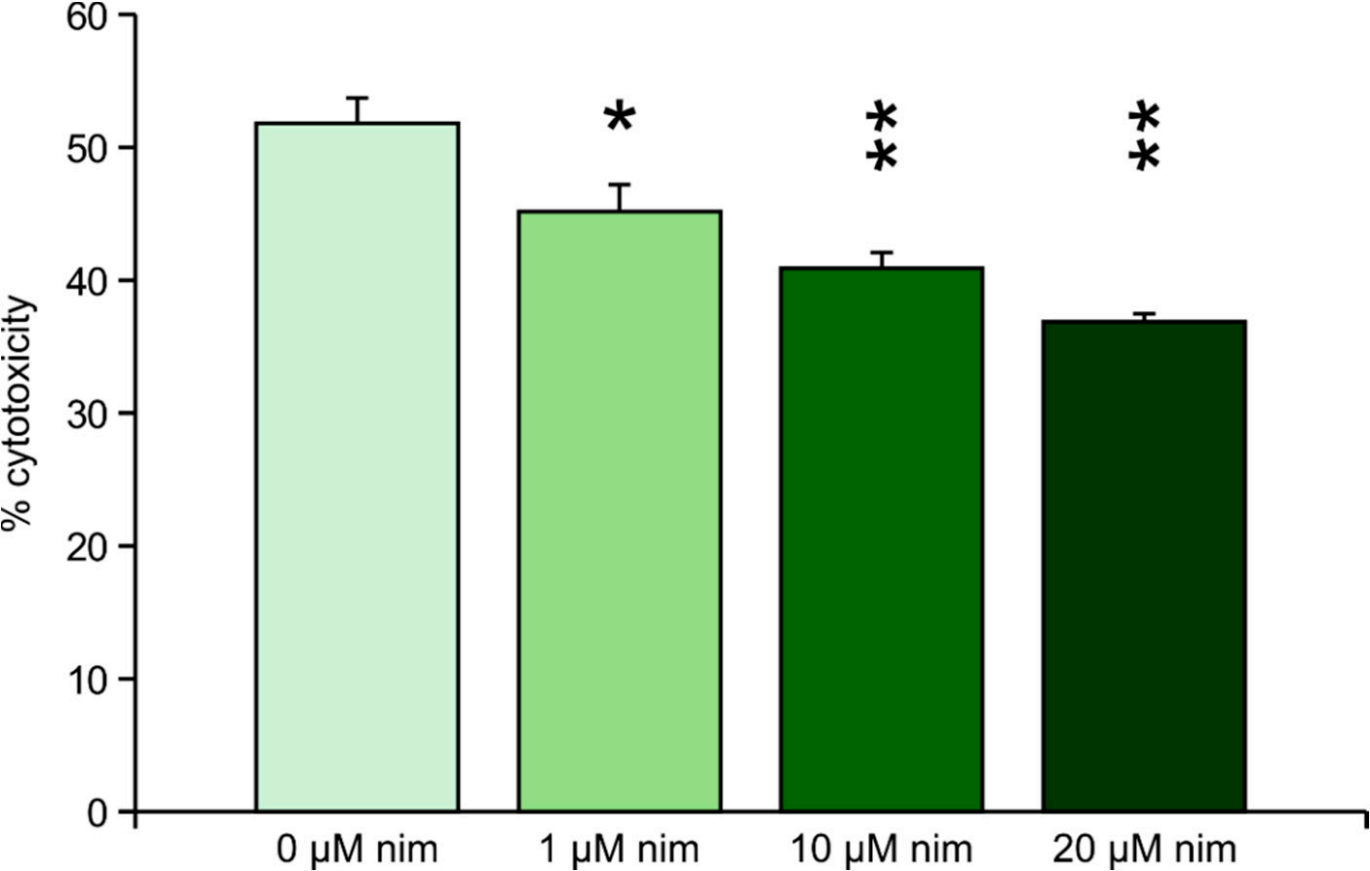
LDH measurement after mechanical stress. Values are given as the mean ± SD (error bars) of one representative out of at least three biologically independent experiments. Nim: nimodipine; single asterisk: *p* ≤ 0.05 compared to non-treated cells; double asterisk: *p* ≤ 0.005 compared to non-treated cells.

Mechanical stress was induced by adding two steel beads (2 mm) to each well of a 24 well-plate of pre-treated or untreated cells, respectively, and shaking the plate at 500 rpm for 30 s. Nimodipine reduced the cytotoxicity from 52% (untreated cells) to 45% (1 μM nimodipine), 40% (10 μM nimodipine) and 37% (20 μM nimodipine). All measured reductions of cytotoxicity were significant (*p* ≤ 0.05), but higher significance (*p* ≤ 0.005) was calculated for 10 and 20 μM nimodipine (Figure 3).

### 2.2. Necrosis and Apoptosis Analyses

**Figure 4:**
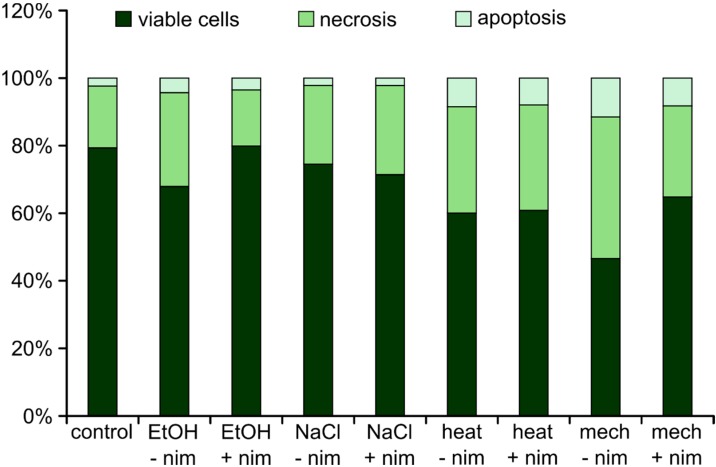
Flow cytometry. Values are given in % of total cells. –nim = without (w/o) nimodipine; +nim = 20 μM nimodipine; EtOH = 2% ethanol; NaCl = 150 mM NaCl; heat = 6 h, 42 °C; mech = shaking with steel beads.

To analyze if the cells undergo necrotic or apoptotic cell death, PI/Annexin staining was analyzed by flow cytometry. Nimodipine non-treated and pre-treated (20 μM) Neuro2a cells were challenged with 2% EtOH, 150 mM NaCl, 6 h at 42 °C or mechanical treatment, respectively. Untreated cells served as the control. In general, all of these stressors induce necrosis more likely than apoptosis (Figure 4).

### 2.3. Live/Dead Staining with FDA and PI

**Figure 5:**
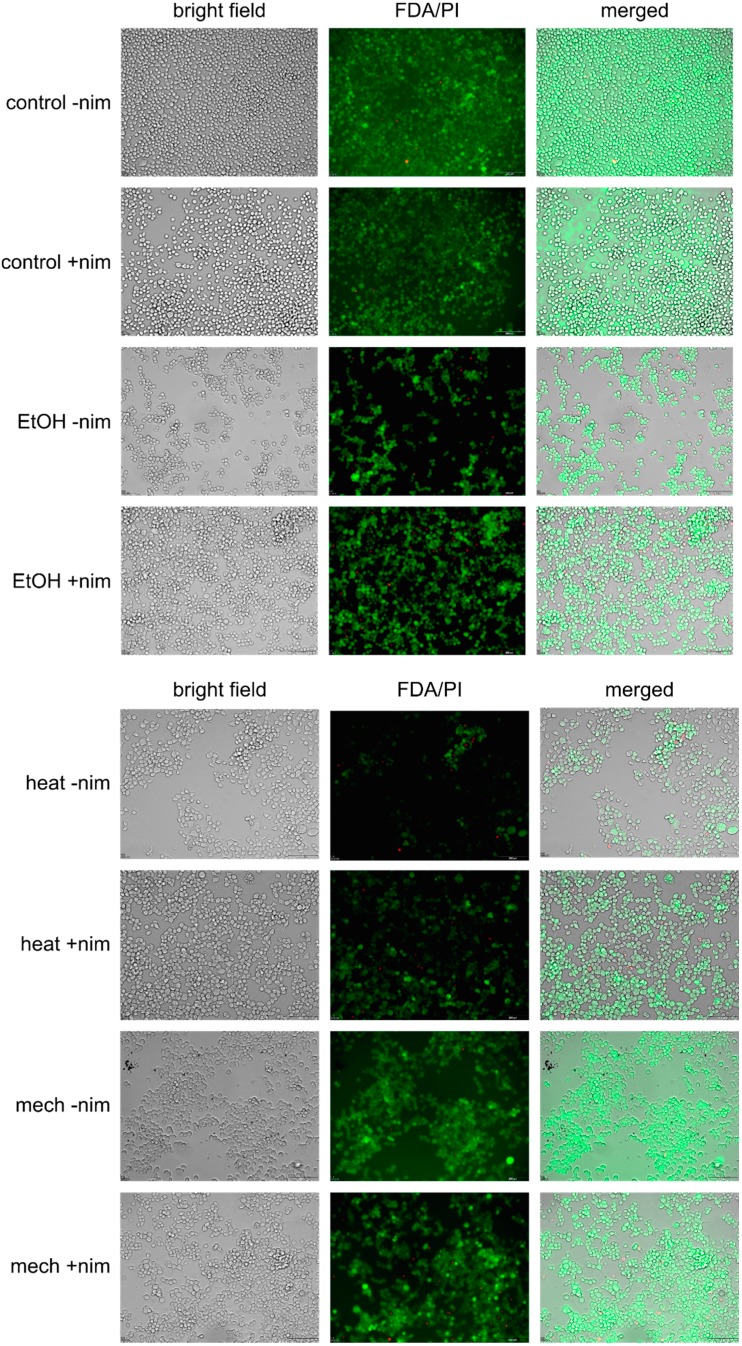
Live/dead staining with fluorescein diacetate (FDA) and propidium iodide (PI). –nim = w/o nimodipine; +nim = 20 μM nimodipine; EtOH = 2% ethanol; heat = 6 h, 42 °C; mech = shaking with steel beads; scale bar = 200 μm.

Live/dead staining was performed using FDA (fluorescein diacetate) and PI (propidium iodide). FDA is metabolized by viable cells, which leads to fluorescein fluorescence. PI can only pass membranes of non-viable cells. Nimodipine non-treated and pre-treated (20 μM) Neuro2a cells were challenged with 2% EtOH, 150 mM NaCl, 6 h at 42 °C or mechanical treatment, respectively. Non-stressed cells served as the control. In general, less cells were detected after stress in samples that were not pre-treated with nimodipine. In all samples, rather few dead cells were visible. Furthermore, more cell-cell connections could be detected in nimodipine pre-treated samples (Figure 5 and Figure S2).

## 3. Discussion 

Nimodipine is a dihydropyridine calcium antagonist with a long history and a good safety profile. Its neuroprotective effect has been shown in several clinical trials [2,8,14] and animal experiments [4,5]. Nimodipine binds to the α1 subunit of the L-type calcium channel [15] and is rapidly and widely distributed in cerebral tissue [16]. The function of nimodipine is thought to promote neuroprotection [9] by preventing Ca^2+^ overload induced by glutamate release, at least in cerebral ischemia [16]. Lipton showed in 1999 that excessive intracellular Ca^2+^ concentrations, excitotoxic glutamate activity and the production of ROS (reactive oxygen species) contribute to neuronal cell death [17]. Changes in intracellular Ca^2+^ concentration also trigger cell death [18]. Ca^2+^ overload in neurons during ischemic cerebral injury has adverse effects followed by brain damage [19]. However, ischemic animal model experiments showed different results concerning nimodipine’s positive effect on the severity of strokes [20]. The conclusion of the Very Early Nimodipine Use in Stroke (VENUS) trial, that nimodipine does not support the hypothesis of a beneficial effect of early nimodipine in stroke patients, may be limited, since nimodipine treatment started within six hours after stroke. Nimodipine was administered orally with a dosage of only 30 mg every six hours [10]. 

The ability of nimodipine to protect against neuronal damage has been described using animal models [9,21].

The neuroprotective mechanisms of nimodipine concerning the better long-term outcome of cranial nerve functions following vestibular Schwannoma surgery [6,22] is unknown. Nimodipine treatment prior to surgery has been shown to be superior to its intraoperative start [23]. In the present study, we systematically analyzed the neuroprotective efficacy of nimodipine on ethanol-, osmotic-, heat- and mechanically-induced cell death to evaluate its possible direct effects on neurons. 

To date, much effort has been put on the understanding of the neuroprotective effect of nimodipine on oxygen-glucose deprivation (OGD)-induced [16,24], bilirubin-induced [25] or NMDA-induced [26] cell death. The neuroprotective capability of nimodipine was also analyzed previously regarding inflammation-mediated degeneration of neurons and showed significantly better results for nimodipine-treated compared with non-treated cell cultures [27]. Neuroprotection by nimodipine was also shown after OGD and trophic withdrawal-induced cell death on nerve growth factor (NGF)-differentiated PC12 neuronal cells [24]. Bilirubin-induced neurotoxicity could be reduced by pre-incubation primary rat cortical neurons with nimodipine, but also by ruthenium red and basilen blue [25]. Most studies concerning the neuroprotective effect of nimodipine deal with OGD-induced cell death [24]. 

Within the model presented in this study, ethanol-, osmotic-, heat- and mechanically-induced stress were analyzed regarding the survival of nimodipine pre-treated Neuro2a cells. The survival of Neuro2a cells was significantly higher when cells were pre-treated with nimodipine prior to stress application, except for osmotic stress. In principle, a higher nimodipine dosage led to higher cell survival concerning ethanol, osmotic and mechanical stress. Significance shows that this is not due to chance. The concentration-dependent potency of nimodipine was also seen in clinical trials, where detectibly higher nimodipine concentrations in the blood serum of patients correlated with better clinical outcomes [28].

Yet, very little is known about the molecular mechanism of the neuroprotection in general. It was observed that estrogens promote neuronal survival and hippocampal neurogenesis via a G protein-coupled receptor (GPR) 30-dependent mechanism [29,30], especially with respect to cognitive functions in Alzheimer’s disease. Carbazoles also contribute to neuroprotection, maybe via their anti-oxidative activity [31]. Furthermore, omega-3 polyunsaturated fatty acids were shown to provide neuroprotection after stroke [32]. Gahm *et al.* showed a significant decrease of degenerated neurons early after trauma linked to nimodipine, but also to colchicine, dexamethasone and tirilazad mesylate [33]. In the same study, the expression of iNOS (inducible nitric oxide synthase) was reduced by nimodipine. A posttraumatic upregulation of iNOS is indicated following brain trauma [34]. iNOS is thought to be involved in inflammatory reactions and may participate in the synthesis of NO in the injured brain [35]. To our knowledge, nothing is known about the molecular mechanisms of neuroprotection concerning nimodipine in particular. 

Here, we show that nimodipine protects cells from ethanol-, heat- and mechanically-induced stress, but not from osmotic stress. Maybe, osmotic stress is too heavy to be reduced by nimodipine, because cells lyse. The most important stressor is certainly mechanical stress, because tumor dissection *per se* induces damage in the surrounding tissue. All analyzed stressors seem to induce necrosis rather than apoptosis. The fact that rather few dead cells were visible in FDA/PI staining may be due to the washing of dead cells when removing the cell culture medium before adding the staining solution. It can be supposed that lower total cell numbers also point to this.

Ethanol is linked to oxidative stress, which contributes to neuronal cell death [17]. All organisms and cells suffer from heat treatment, which is known to induce the synthesis of heat shock proteins (HSPs) [36]. HSPs function as regulators for apoptotic cell death [37]. HSPs are also known to be induced by mechanical stress, at least in the cardiovascular system [38]. Likewise, an activation of p53 is known to be induced by mechanical stress in the cardiovascular system [39]. In mechanically challenged rat cortical neurons, the increase of SIRT1 expession seems to play a role in neuroprotection [40]. Another possible target is the unfolded protein response and/or endoplasmic reticulum stress, respectively, both of which are described with regard to neuronal injury [41].

Regarding the IC_50_ of nimodipine, the analyzed concentrations in this study seem comparatively high. However, we believe that the neuroprotective effect of nimodipine does not necessarily rely on its function as a Ca^2+^ blocking agent, but is rather a side effect. Therefore, we are, at this time point, not interested in other Ca^2+^ blockers, such as nifedipine and others.

In the future, the underlying cellular mechanism of nimodipine concerning its neuroprotective function and its target within signal pathways need to be uncovered for a better understanding of neuroprotection during and after surgery. Therefore, the primary cell culture of neurons and astrocytes could give an insight into the targets of nimodipine.

## 4. Experimental Section

### 4.1. Cell Culture

No institutional permission was required for this study. Cells were treated with 1, 10 or 20 μM or without nimodipine diluted in EtOH absolute 24 h prior to the stress application. The cytotoxicity induced by stress treatment was measured by the amount of LDH that was released into the culture medium 24 h after stress treatment. 

Neuro2a cells were cultured in Dulbecco’s Modified Eagle Medium (DMEM, Life Technologies, Darmstadt, Germany) supplemented with 5% fetal calf serum (FCS, Life Technologies), 1% non-essential amino acids (NEAA, Life Technologies) and penicillin/streptomycin (100 U/mL/100 mg/mL) in cell culture 75-cm^2^ plastic flasks (Greiner, Frickenhausen, Germany) under a humidified atmosphere with 5% CO_2_ at 37 °C. Next, 1 × 10^5^ cells were seeded on 24-well plates (Greiner) and incubated with 1, 10 or 20 μM nimodipine (final concentration), respectively, diluted from a 1000× stock solution in EtOH abs. 24 h prior to stress application. Equal amounts of EtOH were added to non-treated controls. After stress treatment, cells were incubated for another 24 h under humidified atmosphere with 5% CO_2_ at 37 °C. 

Pretests were performed to determine ethanol and NaCl concentrations, as well as incubation time and conditions of mechanical treatment in order to achieve 40% to 60% cell death on average. Thus, changes in cell death induced by nimodipine treatment could be traced.

Cell stress was induced, and the following groups were analyzed:

(1) Ethanol: nimodipine pre-treated and control cells were treated with 1.8% EtOH.
(2) Osmotic stress: nimodipine pre-treated and control cells were treated with 100, 125, 150, 175 or 200 mM NaCl.
(3) Heat: nimodipine pre-treated and control cells have been incubated at 42 °C for 2, 4 or 6 h and then returned to 37 °C until 24 h.
(4) Mechanical stress: pre-treated and control cells were shaken with two 2-mm steel beads at 500 rpm for 30 s. Afterwards, the steel beads were magnetically removed.

All tests were performed as biological triplicates, each as technical triplicates (three wells per sample per plate). Wells were measured 4 times.

### 4.2. LDH Assay

Cytotoxicity was analyzed by using the Cytotoxicity Detection Kit (LDH, Roche, Mannheim, Germany) following the manufacturer’s instructions. In brief, 100 μL freshly prepared reaction mixture were added to each 100 μL of the cell culture medium and incubated for 20 min. Absorbance was measured at 492 nm. Adding 2% Triton X-100 to the cells resulted in totally lysis and served as the positive control (100% cell death).

### 4.3. Necrosis and Apoptosis Analysis

Neuro2a cells were seeded at 1 × 10^6^ in 10-cm dishes and pre-treated with either 20 μM nimodipine or ethanol in the corresponding concentration. After 24 h of pre-treatment, cells were stressed with 1.8% EtOH, 150 mM NaCl, 6 h of 42 °C or shaking with steel beads, respectively. After another 24 h, cells were detached and centrifuged. After washing with PBS, cells were resuspended in 1 mL Annexin V binding buffer (10 mM Hepes, 140 mM NaCl, 205 mM CaCl_2_, pH 7.4). One hundred microliters of cell suspension were incubated with 5 μL FITC Annexin V (BD Biosciences, Heidelberg, Germany) and 10 μL propidium iodide (PI) (Calbiochem, Darmstadt, Germany, 50 μg/mL in PBS) for 15 min at room temperature in the dark. Afterwards, 400 μL Annexin V binding buffer were added, and flow cytometry was performed using FACScan (BD Biosciences).

### 4.4. Live/Dead Staining with FDA and PI

Neuro2a cells were pre-treated with either 20 μM nimodipine or ethanol in the corresponding concentration. After 24 h of pre-treatment, cells were stressed with 1.8% EtOH, 150 mM NaCl, 6 h of 42 °C or shaking with steel beads, respectively. After another 24 h, the medium was removed and staining solution (5 mL DMEM w/o FCS, 8 μL FDA (fluorescein diacetate, Sigma-Aldrich, 5 mg/mL in acetone), 50 μL PI (Calbiochem, 2 mg/mL in PBS)) was added. Cells were incubated 4–5 min at room temperature in the dark. Staining solution was removed and PBS was added. Samples were analyzed using the Olympus IX81 (Olympus, Hamburg, Germany) at 495 nm excitation and detection by the 510–550 nm band filter for FDA and 596 nm excitation and detection by the 663–737 band filter for PI.

### 4.5. Statistical Analysis

Significance within one experimental setup was determined using the one-way ANOVA. The reference for the *p*-values is the nimodipine non-treated sample of the same assay. Statistically significant differences are presented at probability levels of *p* ≤ 0.05.

## 5. Conclusions

Nimodipine has a neuroprotective capability concerning selected types of stressors, particularly with regard to surgery. The mechanisms of neuroprotection by nimodipine remain unknown and need to be investigated in detail by using cell culture models in order to advance preventive medication.

## Supplementary Materials

Supplementary figures can be found at http://www.mdpi.com/1422-0067/15/10/18453/s1.

## Supplementary Files

Supplementary File 1
