# Supplementary material for: Investigation of the Neuroprotective Impact of Nimodipine on Neuro2a Cells by Means of a Surgery-Like Stress Model
Source: Int J Mol Sci. 2014 Oct 14;15(10):18453–65. doi: 10.3390/ijms151018453 (PMC4227225; doi:10.3390/ijms151018453)
Supplement: Supplementary File 1 [file ijms-15-18453-s001.pdf]

Supplementary Information

Figure S1. LDH measurement after osmotic stress. Values are given as the mean ± SD (error bars) of one representative out of at least three biologically independent experiments. Nim = nimodipine.

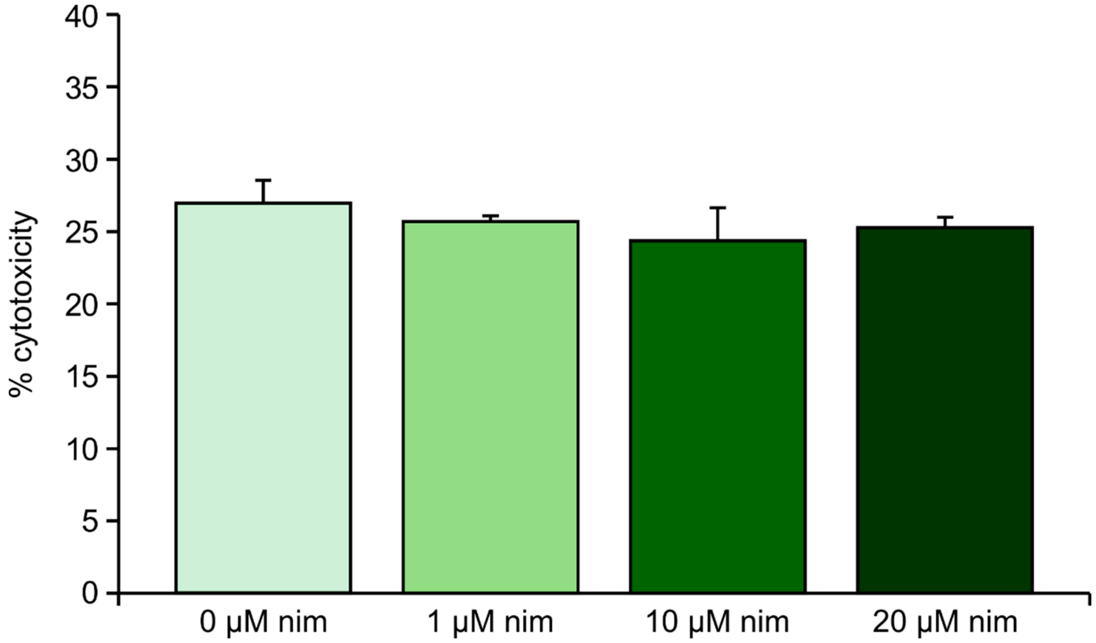

Figure S2. (**a**) Live/dead staining with FDA and PI. enlargement of microscopy pictures (control, EtOH stress) –nim = w/o nimodipine; +nim = 20 µM nimodipine; control = non-stressed cells; EtOH = 2% ethanol; (**b**) Live/dead staining with FDA and PI. enlargement of microscopy pictures (control, EtOH stress) –nim = w/o nimodipine; +nim = 20 µM nimodipine; heat = 6 h, 42 °C; mech = shaking with steel beads; and (**c**) Live/dead staining with FDA and PI enlargement of microscopy pictures (control, EtOH stress) –nim = w/o nimodipine; +nim = 20 µM nimodipine; NaCl = 150 mM NaCl; scale bar = 200 µm.

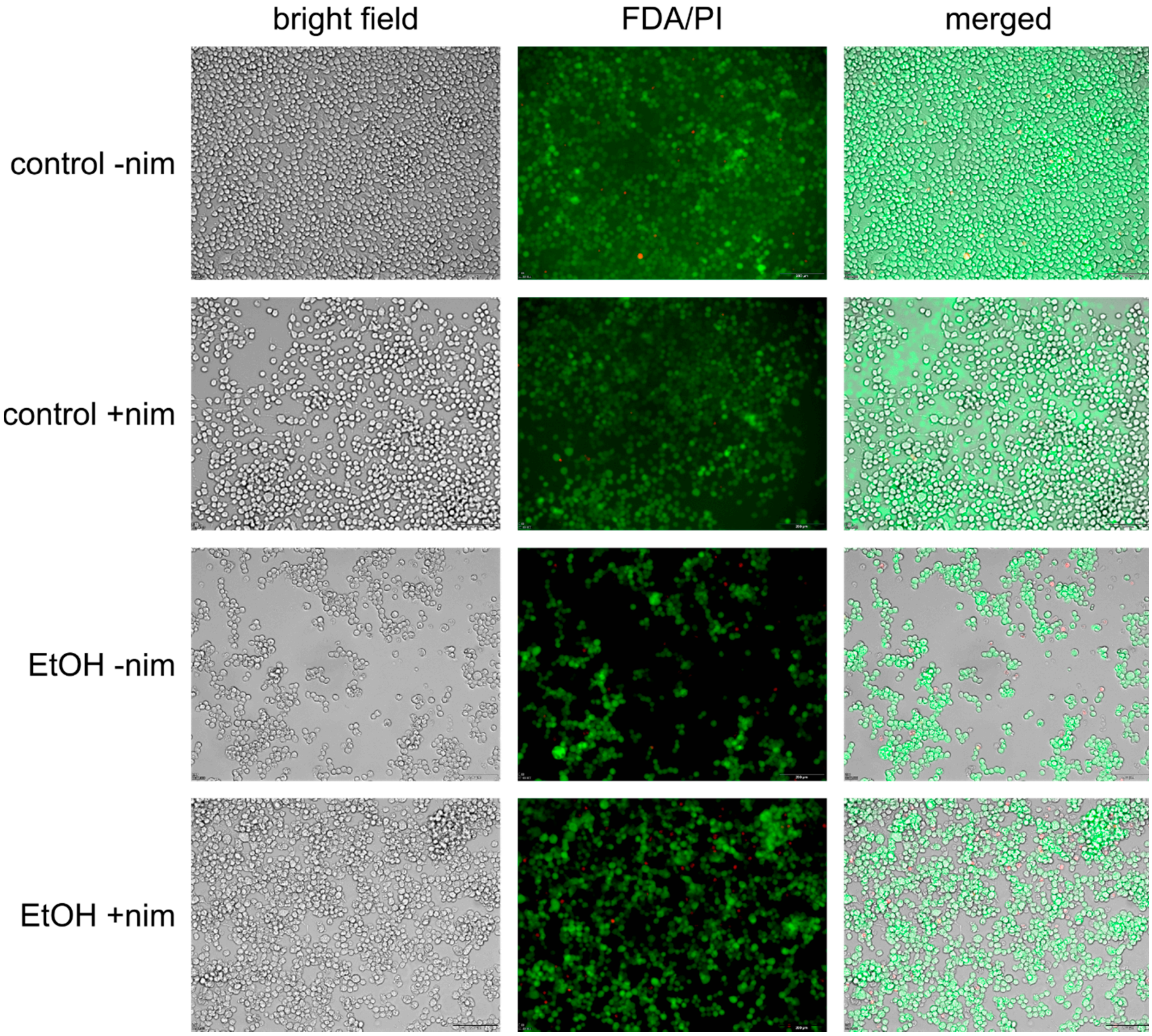

(**a**)

Figure S2. *Cont.*

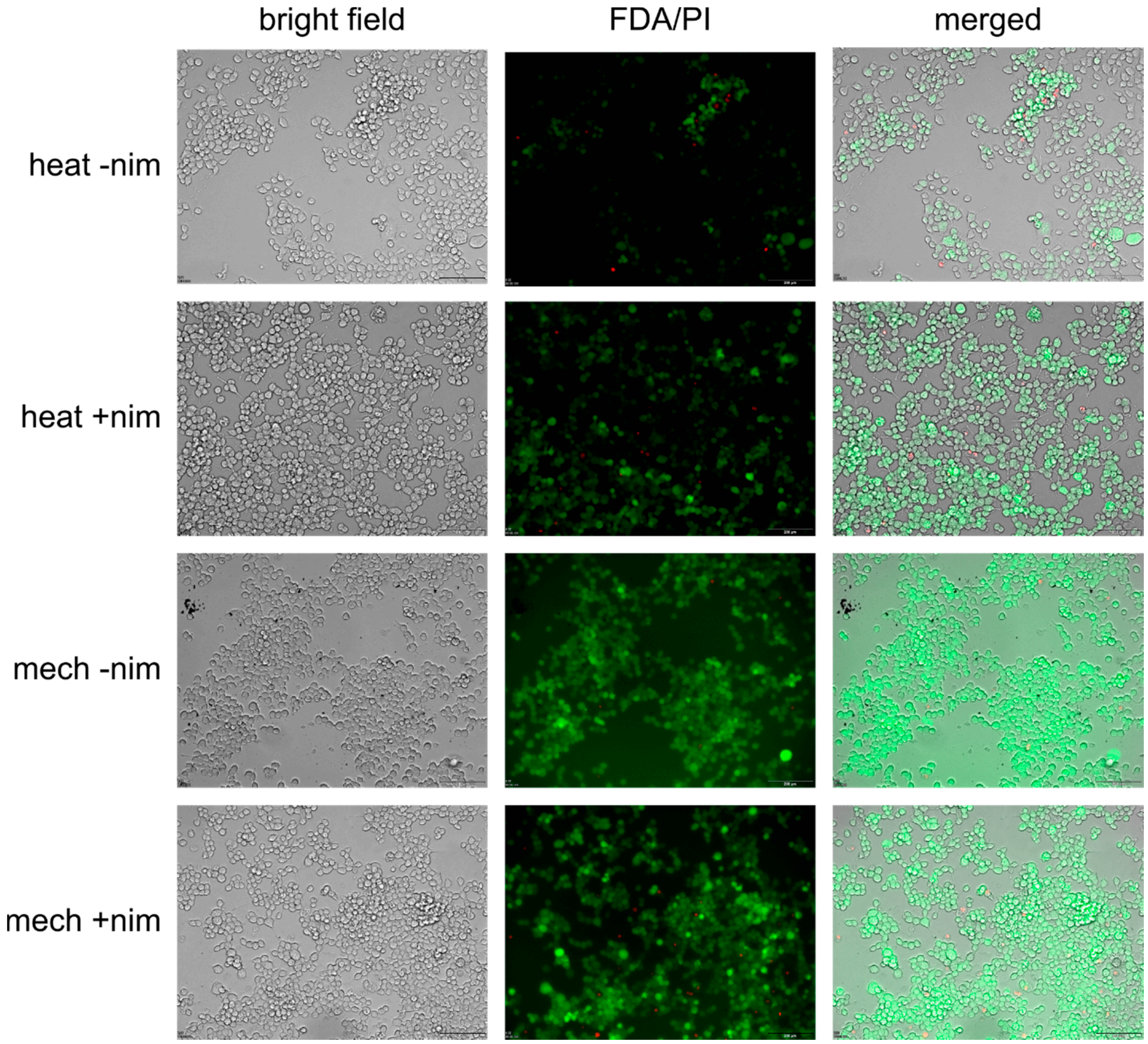

(**b**)

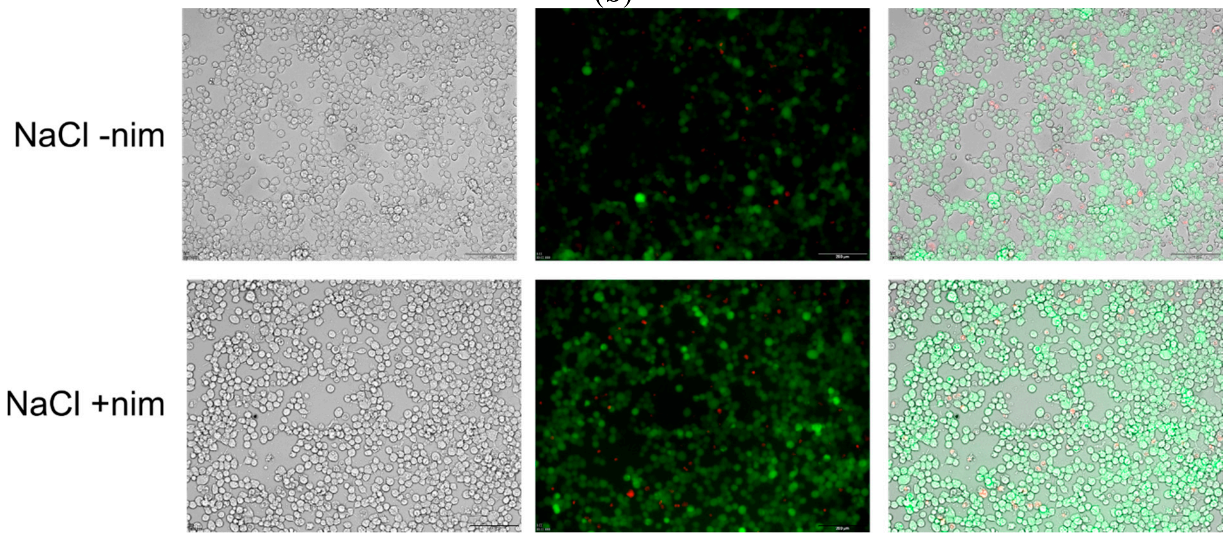

(**c**)
